# Robotic Approach to Right-Sided Hiatal Hernia Involving the Ascending and Transverse Colon, Duodenum, and Distal Stomach: A Case Report

**DOI:** 10.70352/scrj.cr.26-0190

**Published:** 2026-07-16

**Authors:** Woo Jun Kim, Dongwon Lim, Joohyung Son, Bong Soo Son, Si-Hak Lee, Sun-Hwi Hwang, Jae Hun Chung

**Affiliations:** 1 29th Regiment, Korea Army Training Center, Nonsan, South Chungcheong Province, Republic of Korea Army; 2 Division of Gastrointestinal Surgery, Department of Surgery, Pusan National University Yangsan Hospital, Yangsan, South Gyeongsang Province, Republic of Korea; 3 Department of Thoracic Surgery, Pusan National University Yangsan Hospital, Yangsan, South Gyeongsang Province, Republic of Korea; 4 School of Medicine, Pusan National University, Yangsan, South Gyeongsang Province, Republic of Korea; 5 Research Institute for Convergence of Biomedical Science and Technology, Pusan National University Yangsan Hospital, Yangsan, South Gyeongsang Province, Republic of Korea

**Keywords:** case reports, robotic surgery, robotic hernia repair, right-sided hiatal hernia, paraesophageal hernia, hiatal hernia

## Abstract

**INTRODUCTION:**

Cases of hiatal hernia (HH) involving the distal stomach, duodenum, and ascending and transverse colon are exceptionally rare and present significant surgical challenges owing to severe anatomical distortion. Although minimally invasive surgery is the gold standard for HH repair, the application of a robotic approach to this specific complex configuration involving distal gastric and duodenal herniations has not been previously reported. Here, we present the case of such a defect that was managed successfully using a robotic platform.

**CASE PRESENTATION:**

A 36-year-old female presented with worsening vomiting and an inability to tolerate oral intake for 1 week prior to admission. Her history included 2 recent pregnancies and deliveries within the last 2 years, which likely precipitated the symptoms due to increased intra-abdominal pressure. CT of the abdomen and pelvis revealed a massive HH. The herniated contents included the lower gastric body, antrum, duodenal bulb, and ascending and transverse colon, all of which were displaced into the right hemithorax via a defect in the right crus of the diaphragm. The patient underwent a total robotic repair using the da Vinci Xi surgical system (Intuitive Surgical, Sunnyvale, CA, USA). The procedure involved careful reduction of the herniated viscera and meticulous mediastinal dissection of the hernial sac using a soft coagulation technique. The robotic platform facilitated the identification and preservation of the anterior and posterior vagal trunks. The crural defect was repaired using nonabsorbable sutures (Ethibond 2-0; Ethicon, Raritan, NJ, USA). Owing to the emergency setting and extensive anatomical distortion, anterior gastropexy was performed instead of fundoplication to secure the stomach and prevent recurrence. Obstructive symptoms resolved immediately postoperatively. She successfully transitioned to oral intake. The chest tube was removed on POD 5, and she was discharged on day 7 without complications.

**CONCLUSIONS:**

This case demonstrates the feasibility and safety of a completely robotic approach for repairing complex HH involving the distal stomach, duodenum, and ascending and transverse colon. The robotic platform’s superior 3D visualization and wrist articulation are distinct advantages for performing precise dissection in the confined mediastinal space, ensuring safe reduction and preservation of critical structures such as the vagal nerves.

## Abbreviation

HH
: hiatal hernia

## INTRODUCTION

HH is a prevalent anatomical defect involving the protrusion of abdominal viscera into the mediastinum, and is classified into 4 distinct types based on the extent and nature of herniation.^1)^ Although asymptomatic cases are often managed conservatively, symptomatic paraesophageal hernias—particularly Type IV defects involving other intra-abdominal organs—necessitate prompt surgical intervention to prevent life-threatening complications such as obstruction, strangulation, or gastric volvulus.^2)^ Since the shift from open surgery, minimally invasive surgery has become the gold standard for repair, significantly reducing perioperative morbidity and hospital stay.^3)^ Recently, robotic platforms have further refined these procedures, offering superior 3D visualization and enhanced wrist articulation. These technical advantages are particularly crucial in complex repairs, potentially lowering recurrence rates and overcoming the limitations of conventional laparoscopy in challenging anatomical fields.

Among complex hiatal defects, herniation involving the distal stomach, duodenum, and ascending and transverse colon is exceptionally rare and presents unique surgical challenges due to severe anatomical distortion and the risk of incarceration.^4)^ Although limited literature exists regarding open or laparoscopic management of this condition, the application of a robotic approach for this specific distal involvement has not yet been reported. In this study, we present a rare case of HH involving the distal stomach, duodenum, and ascending and transverse colon, precipitated by pregnancy-related increased intra-abdominal pressure, which was successfully managed using a robotic approach. We discuss the safe reduction of the herniated viscera and highlight the distinct advantages of robotic technology in addressing anatomically complex and unprecedented presentations.

## CASE PRESENTATION

The patient was a 36-year-old female (height: 165 cm; weight: 59 kg; BMI: 21.67 kg/m^2^) with a history of 2 recent deliveries (March 2024 and March 2025). She underwent a total thyroidectomy for thyroid cancer in May 2025. Her current medications included levothyroxine 150 µg, 1 tablet daily; calcium carbonate 1250 mg with cholecalciferol 25 µg, 1 tablet daily; and calcitriol 0.25 µg, 1 tablet daily. At 20 years of age, a right-sided HH was incidentally suspected on chest radiography, and no medical follow-up was pursued due to the absence of symptoms.

After her second delivery, her vomiting gradually worsened, and 1 week prior to presentation, she was unable to tolerate oral water intake with projectile vomiting and epigastric discomfort, prompting an emergency department visit to a nearby secondary hospital.

Physical examination revealed signs of dehydration, but no abdominal tenderness or rebound tenderness. Laboratory results revealed a white blood cell count of 6370/µL (segmental neutrophils, 70.7%), lactic acid of 1.7 mmol/L, and a high-sensitivity C-reactive protein level of 0.72 mg/dL.

CT of the abdomen and pelvis revealed an HH. The herniated contents included the lower gastric body, antrum, duodenal bulb, and ascending and transverse colon, all displaced into the right hemithorax through a defect in the right crus of the diaphragm (**Fig. 1**).

**Fig. 1:**
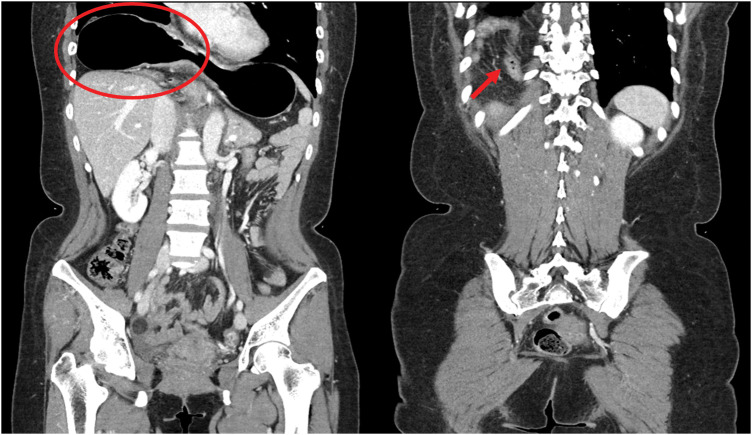
Abdominal and pelvic CT on a visit to the emergency department. Herniation of the stomach, duodenum, and ascending and transverse colon into the right hemithorax is observed, resulting in collapse of the right lung.

The patient underwent robotic HH repair under general anesthesia using the da Vinci Xi Surgical System (Intuitive Surgical, Sunnyvale, CA, USA). After adequate liver traction was established, the herniated stomach and other herniated viscera were carefully reduced into the abdominal cavity. The hernia sac was clearly identified and meticulously dissected using a soft coagulation technique, as described in the established robotic approaches.^5)^ Circumferential mediastinal dissection of the hernia sac was performed, followed by extensive mobilization of the distal esophagus to achieve sufficient intra-abdominal esophageal length.^6)^ Throughout the procedure, particular attention was paid to the preservation of the anterior and posterior vagal trunks (**Fig. 2A**–**2D**). According to intraoperative findings, after dissection of the hernia sac, the lower esophagus and vagus nerve trunk were identified; there was no tension that could be caused by a short esophagus.

**Fig. 2:**
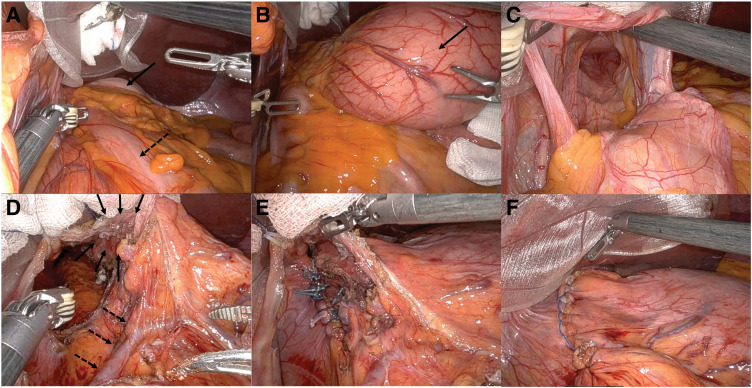
Intraoperative findings. (**A**) The stomach (solid arrow) and colon (dashed arrow) are herniated through the right crus of the diaphragm. (**B**) The stomach (solid arrow) is gently retracted using robotic forceps, and the hernia is successfully reduced. (**C**) The hernia sac is identified. (**D**) Retracted vagal trunk and lower esophagus (solid arrows) and left gastric artery and vein (dashed arrow). (**E**) The right and left diaphragmatic crura are approximated with interrupted nonabsorbable sutures (Ethibond 2-0; Ethicon, Raritan, NJ, USA). (**F**) With a nonabsorbable interlocking suture (V-Loc 3-0; Medtronic, Minneapolis, MN, USA), the lesser omentum is closed.

Crural repair was completed with interrupted nonabsorbable sutures (Ethibond 2-0; Ethicon, Raritan, NJ, USA) and reinforced with a continuous barbed nonabsorbable suture (V-Loc 3-0; Medtronic, Minneapolis, MN, USA). The lesser omentum was restored to its native anatomical configuration using a nonabsorbable interlocking suture (V-Loc 3-0) (**Fig. 2E** and **2F**). Given the presumed long-standing nature of the HH (at least 15 years), chronic atelectasis of the adjacent lung parenchyma was observed on the preoperative CT scan. During the robotic dissection of the large hernia sac, the parietal pleura was entered, leading to an intraoperative capnothorax as CO_2_ gas migrated into the thoracic cavity. However, no lung parenchymal injury was identified. Postoperatively, persistent radiologic findings consistent with a pneumothorax were observed, likely due to a combination of this capnothorax and delayed lung re-expansion after chronic compression. To facilitate the expansion of the long-collapsed lung and ensure respiratory stability, a chest tube was inserted on POD 1 following consultation with the Department of Thoracic Surgery at Pusan National University Yangsan Hospital.

The patient’s obstructive symptoms resolved immediately, and she was switched to a liquid diet. The chest tube was removed on POD 5. The patient tolerated diet advancement well and was discharged on POD 7 after resumption of a regular diet (**Fig. 3**).

**Fig. 3:**
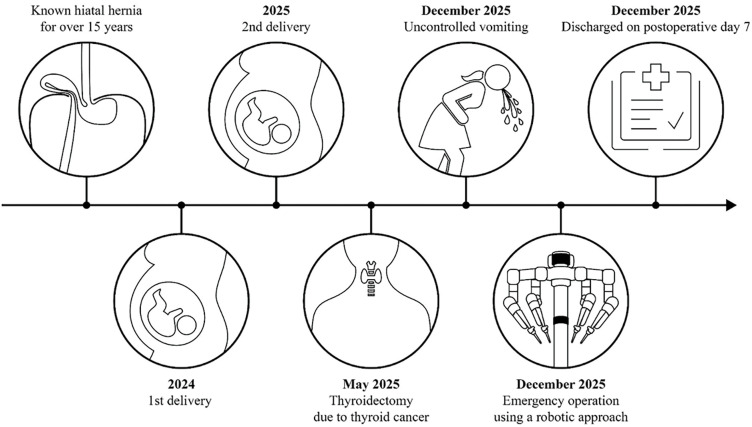
Clinical timeline of a long-standing HH becoming symptomatic after repeated pregnancies. Schematic of the patient’s clinical course. The patient had an HH for >15 years without significant symptoms. After the first delivery in 2024 and the second delivery in 2025, the patient developed progressive gastrointestinal symptoms. In May 2025, the patient underwent thyroidectomy for thyroid cancer. In December 2025, she presented with uncontrolled vomiting due to an acute exacerbation of an HH that required emergency surgical repair using a robotic approach. The postoperative course was uneventful, and the patient was discharged on POD 7. HH, hiatal hernia

## DISCUSSION

Right-sided HH is anatomically distinct from the more common Type I–IV HH. It occurs through a defect in the right crus of the diaphragm, allowing intra-abdominal organs to herniate into the thoracic cavity. In the present case, the gastric antrum, duodenum, ascending and transverse colon, and omentum herniated into the thoracic cavity, resulting in severe obstruction. Based on the CT findings and intraoperative observations, there was no evidence of tissue ischemia or necrosis due to incarceration; therefore, it was presumed that the absence of abdominal tenderness on physical examination at the time of emergency department presentation at Pusan National University Yangsan Hospital was consistent with these findings.

In this patient, an HH that had remained asymptomatic for at least 15 years subsequently became symptomatic with obstructive features, which were presumed to be associated with repeated pregnancies and childbirth. Two pregnancies over a 2-year period, accompanied by weight gain and markedly increased intra-abdominal pressure associated with vaginal delivery, may have induced mechanical alterations at the gastroesophageal junction, thereby precipitating the onset.^7)^

Since Cuschieri et al. first introduced laparoscopic HH repair in 1992, the laparoscopic approach has gradually gained widespread acceptance and is currently employed in most cases.^8,9)^ With advances in robotic platforms, the use of robotic surgery has increased, and several studies have suggested potential technical advantages over conventional laparoscopy. The robotic platform provided superior visualization for the dissection of the hernia sac, particularly in the narrow mediastinal space.^3)^ The magnified 3D visualization and multiarticulated instruments facilitated meticulous dissection within the deep mediastinum. This was particularly vital for identifying and preserving the vagal trunks and lower esophagus (**Fig. 2D**), which were displaced and adherent to the enlarged hernia sac. The ability to perform fine dissections parallel to these nerves minimizes the risks of neuropraxia and transection.

The transthoracic approach offers several advantages, including superior visualization and excellent accessibility to the esophagus. Additionally, a combined approach using video-assisted thoracoscopic surgery and laparoscopy has been increasingly employed.^10)^ However, a transthoracic approach necessitates 1-lung ventilation, which carries a substantially greater anesthetic burden—particularly in emergency cases or elderly or comorbid patients—and this consideration should not be overlooked when selecting the surgical approach. Moreover, regarding suturing ease, the articulated instrumentation of robotic surgery largely mitigates the influence of the access route on technical precision, rendering the abdominal versus thoracic approach less relevant in this regard. In the present case, multiple intra-abdominal organs had herniated simultaneously; therefore, the transabdominal approach provided more advantages for assessing the viability of these organs and ensuring their proper repositioning. Furthermore, in performing gastropexy and suturing the lesser omentum, this approach provided a more favorable camera angle. Although direct comparative evidence between transthoracic and transabdominal robotic HH repair remains limited, this represents an area warranting future investigation.

We used a soft coagulation technique for sac excision. Unlike high-energy devices that pose a risk of collateral thermal damage, soft coagulation enables pinpoint hemostasis near sensitive structures.^11,12)^ Furthermore, robotic articulation simplifies the closure of the wide hiatal defect. Suturing at the superior apex of the hiatus was performed with precision using barbed sutures (V-Loc), ensuring secure crural approximation under high tension without tissue tearing.

The decision to perform gastropexy rather than mesh reinforcement and Nissen fundoplication was a strategic choice driven by the emergent nature of the case and extensive anatomical distortion. Although fundoplication is the standard for preventing reflux in elective repairs, the risk of a “short esophagus” and the potential to create a high-pressure zone in an ischemic stomach outweighed the benefits; moreover, intraoperative endoscopic verification of wrap integrity was not feasible in this emergency setting. Mesh reinforcement was similarly avoided, as introducing a prosthetic foreign body into an acutely distorted surgical field carries significant risks of infection and visceral erosion, and the robotic platform’s precision enabled a robust primary crural closure using a combination of interrupted nonabsorbable and barbed sutures without prosthetic augmentation—an approach supported by long-term randomized evidence showing no recurrence benefit of mesh over suture repair alone.^13)^ Gastropexy serves as a safer and more effective alternative to stomach fixation to prevent acute gastric volvulus or HH recurrence while avoiding complications associated with over-tightening the hiatus in an acute setting.^14)^

At the 6-month postoperative follow-up, CT demonstrated no evidence of residual or recurrent HH, confirming the durability of the repair (**Fig. 4A** and **4B**). The patient reported no episodes of nausea or vomiting throughout the postoperative period and described a marked improvement in oral intake, tolerating a regular diet without difficulty or discomfort. Overall patient satisfaction was excellent, reflecting both the functional success and the QOL benefit achieved by this operative approach.

**Fig. 4:**
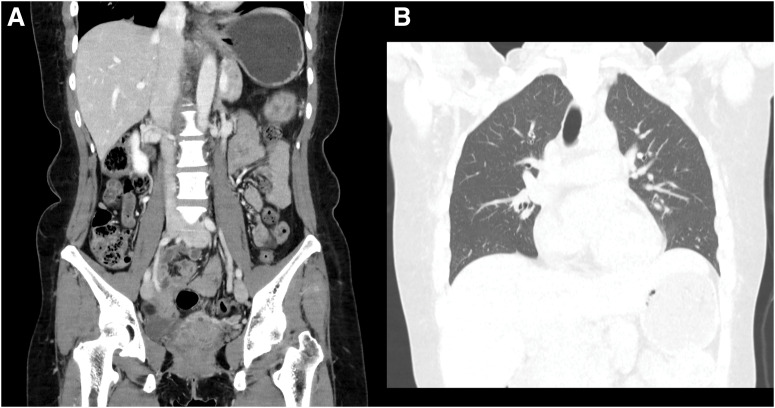
Abdominopelvic and chest CT 6 months after surgery. (**A**) There is no evidence of residual or recurrent HH. (**B**) The lung parenchyma showed complete recovery from atelectasis, with no evidence of capnothorax. HH, hiatal hernia

However, the robotic approach is not without limitations. Laparoscopic HH repair remains more cost-effective than the robotic approach, with no statistically significant differences in operative time, intraoperative complications, length of stay, or readmission rates between the 2 approaches.^15)^ Furthermore, depending on the robotic platform employed, the absence of haptic feedback represents a notable disadvantage compared with conventional laparoscopy, in which tactile force feedback allows the surgeon to perceive tissue resistance in real time. The lack of force feedback during robotic HH reduction may increase the risk of inadvertent tissue injury, particularly when manipulating chronically incarcerated or potentially ischemic herniated contents, where tissue fragility may not be adequately appreciated through visual cues alone.

The apparent pros and cons of the robotic platform observed in this report—including enhanced precision, improved visualization, and facilitation of complex dissection—require validation through larger prospective studies or comparative series before broader conclusions regarding its superiority over conventional laparoscopic approaches can be established.

## CONCLUSIONS

In conclusion, this case illustrates the technical feasibility of a completely robotic approach for repairing an exceptionally rare HH involving the distal stomach, duodenum, and ascending and transverse colon. In this patient, the robotic platform facilitated safe reduction and meticulous dissection of the hernial sac within the confined mediastinal space, enabling precise identification and preservation of the vagal trunks. Although conclusions regarding superiority or generalizability cannot be drawn from a single case, this experience suggests that robotic repair may be a valuable surgical option for selected patients with complex hiatal anatomy. Further accumulation of cases and comparative studies are warranted to better define their roles in rare clinical scenarios.

## DECLARATIONS

### Use of artificial intelligence tools

Language polishing was assisted by Claude (Anthropic PBC, San Francisco, CA, USA).

### Availability of data and materials

The datasets generated and/or analyzed during the current study are not publicly available due to institutional policies on patient privacy and data protection, but are available from the corresponding author upon reasonable request, subject to approval by the Institutional Review Board of Pusan National University Yangsan Hospital.

## References

[1] Dean C, Etienne D, Carpentier B, et al. Hiatal hernias. Surg Radiol Anat 2012; 34: 291–9. 22105688 10.1007/s00276-011-0904-9

[2] Sfara A, Dumitrascu DL. The management of hiatal hernia: an update on diagnosis and treatment. Med Pharm Rep 2019; 92: 321–5. 31750430 10.15386/mpr-1323 PMC6853045

[3] O’Connor SC, Mallard M, Desai SS, et al. Robotic versus laparoscopic approach to hiatal hernia repair: results after 7 years of robotic experience. Am Surg 2020; 86: 1083–7. 32809844 10.1177/0003134820943547

[4] Takeuchi N, Nomura Y. Paraesophageal hernia with incarceration of the gastric antrum and duodenal bulb: a case report. BMC Res Notes 2013; 6: 451. 24207166 10.1186/1756-0500-6-451 PMC3835393

[5] Yamashita Y, Tatsubayashi T, Okumura K, et al. Robotic radical distal gastrectomy for gastric cancer using the soft coagulation scissors technique. J Robot Surg 2023; 17: 605–11. 36169804 10.1007/s11701-022-01459-w

[6] Andolfi C, Jalilvand A, Plana A, et al. Surgical treatment of paraesophageal hernias: a review. J Laparoendosc Adv Surg Tech A 2016; 26: 778–83. 27398823 10.1089/lap.2016.0332 PMC6445204

[7] Lee YY, McColl KE. Disruption of the gastroesophageal junction by central obesity and waist belt: role of raised intra-abdominal pressure. Dis Esophagus 2015; 28: 318–25. 24575877 10.1111/dote.12202

[8] Cuschieri A, Shimi S, Nathanson LK. Laparoscopic reduction, crural repair, and fundoplication of large hiatal hernia. Am J Surg 1992; 163: 425–30. 1532701 10.1016/0002-9610(92)90046-t

[9] Draaisma WA, Gooszen HG, Tournoij E, et al. Controversies in paraesophageal hernia repair; a review of literature. Surg Endosc 2005; 19: 1300–8. 16151684 10.1007/s00464-004-2275-3

[10] Zaman JA, Lidor AO. The optimal approach to symptomatic paraesophageal hernia repair: important technical considerations. Curr Gastroenterol Rep 2016; 18: 53. 27595155 10.1007/s11894-016-0529-6

[11] Uyama I, Kanaya S, Ishida Y, et al. Novel integrated robotic approach for suprapancreatic D2 nodal dissection for treating gastric cancer: technique and initial experience. World J Surg 2012; 36: 331–7. 22131088 10.1007/s00268-011-1352-8

[12] Kinoshita T, Sato R, Akimoto E, et al. Reduction in postoperative complications by robotic surgery: a case-control study of robotic versus conventional laparoscopic surgery for gastric cancer. Surg Endosc 2022; 36: 1989–98. 33844086 10.1007/s00464-021-08483-1 PMC8847173

[13] Analatos A, Håkanson BS, Ansorge C, et al. Hiatal hernia repair with tension-free mesh or crural sutures alone in antireflux surgery: a 13-year follow-up of a randomized clinical trial. JAMA Surg 2024; 159: 11–8. 37819652 10.1001/jamasurg.2023.4976 PMC10568445

[14] Dallemagne B, Quero G, Lapergola A, et al. Treatment of giant paraesophageal hernia: pro laparoscopic approach. Hernia 2018; 22: 909–19. 29177588 10.1007/s10029-017-1706-8

[15] Gonçalves-Costa D, Barbosa JP, Quesado R, et al. Robotic surgery versus laparoscopic surgery for anti-reflux and hiatal hernia surgery: a short-term outcomes and cost systematic literature review and meta-analysis. Langenbecks Arch Surg 2024; 409: 175. 38842610 10.1007/s00423-024-03368-y PMC11156741

